# Exploring the Relationships Between Clinical Examination Findings, Subjective Reported Symptoms and Objective Nasal Patency Measures in Nasal Obstruction: A Baseline NAIROS Sub‐Study Analysis

**DOI:** 10.1111/coa.14221

**Published:** 2024-09-08

**Authors:** Pavithran Maniam, Alison Bray, Michael Drinnan, Tony Fouweather, M. Dawn Teare, Sean Carrie, James O'Hara

**Affiliations:** ^1^ Ear, Nose & Throat Department Newcastle Upon Tyne Hospitals NHS Foundation Trust Newcastle upon Tyne UK; ^2^ Northern Medical Physics and Clinical Engineering Newcastle Upon Tyne Hospitals NHS Foundation Trust Newcastle upon Tyne UK; ^3^ Translational and Clinical Research Institute Newcastle University Newcastle upon Tyne UK; ^4^ Population Health Sciences Institute Newcastle University Newcastle upon Tyne UK; ^5^ Biostatistics Research Group Newcastle University Newcastle upon Tyne UK

**Keywords:** anterior rhino‐meter, anterior rhinoscopy, double ordinal airway subjective scale, objective nasal patency measure, peak nasal inspiratory flow, rhinometry, rhino‐spirometer

## Abstract

**Background:**

The role of objective nasal airflow measures using peak nasal inspiratory flow (PNIF) and rhinospirometry in supporting clinical examination findings when offering patients septoplasty remain undefined.

**Objective:**

To explore the baseline relationships between clinical examination findings, subjective reported symptoms and objective nasal patency measures in nasal obstruction.

**Methods:**

This is a sub‐study of the NAIROS trial. Participants with nasal obstruction secondary to septal deviation were included in this NAIROS sub‐study. The side of septal deviation, enlargement of inferior turbinate (IT), the need for IT reduction if septoplasty was being performed, the area of septum deflecting into the airway and observer rated airway block (ORAB–arbitrarily divided by <50% and >50% blockage) were assessed by clinicians. The subjective score of nasal obstruction was assessed using the Double Ordinal Assessed Subjective Scale (DOASS). Objective nasal patency measures (e.g., nasal partitioning ratio, [NPR] and PNIF) were measured using PNIF and rhinospirometry.

**Results:**

The mean NPR for left‐sided, both‐sided and right‐sided septal deviation was −0.35, −0.02 and 0.51, respectively (*p* < 0.001). There was very weak correlation between the requirement for IT reduction and PNIF change (0.13, *p* < 0.01). There was no difference in mean PNIF (94 L/min vs. 93 L/min) and mean DOASS (0.33 vs. 0.38) for participants with ORAB rated <50% and >50%. The mean NPR for participants with ORAB >50% was higher than for those with ORAB <50% (0.51 vs. 0.41, *p* = 0.002). There was strong correlation between the DOASS and NPR (+0.737, *p* < 0.001). The mean DOASS score for right‐sided, both‐sided and left‐sided septal deviation was 0.32, 0.05 and −0.29, respectively (*p* < 0.001).

**Conclusion:**

This study identified strong relationships between the clinician rated side of septal deflection, the patient reported DOASS and the objective NPR measurements. NPR and the clinician rated degree of airway blockage were concordant.

**Summary**

- Septoplasty is a common surgery to relieve nasal obstruction secondary to a deviated septum
- The role of baseline objective nasal airflow measures and subjective symptoms in supporting clinical examination findings in septal deviation remain undefined.
- Clinician rated side of septal deflection correlates strongly with subjective patient reported symptoms on DOASS
- Clinician rated side of septal deflection correlates strongly with the objective NPR measurements.
- NPR and the clinician rated degree of airway blockage were concordant.

## 1 Introduction

Septoplasty is a common operation to relieve nasal obstruction secondary to a deviated septum [1, 2]. Septoplasty is frequently performed in conjunction with inferior turbinate (IT) reduction, to improve nasal air flow. Approximately 17 000 septoplasties were performed annually between 2012 and 2019 in National Health Service (NHS) hospitals across England [3]. In the United States, approximately 250 000 septoplasties are performed every year [4].

Evidence suggests that septoplasty provides objective and functional benefit to patients with nasal obstruction, by improving the patency of the nasal airway [5, 6, 7]. Despite septoplasty being a common surgical procedure to correct nasal deviation and obstruction, patient selection criteria for septoplasty remain undefined and patients are often offered surgery based on the clinical history and subjective assessment of nasal septal deviation using anterior rhinoscopy or nasendoscopy [7, 8]. The role of objective nasal airflow measures using peak nasal inspiratory flow (PNIF) and rhinospirometry in supporting clinical examination findings when selecting patients for septoplasty are of limited utility [9, 10].

The recent Nasal Airways Obstruction Study (NAIROS)—an open label multicentred randomised controlled trial of septoplasty versus medical management—evaluated the clinical and cost‐effectiveness of septoplasty [11]. The NAIROS clinical trial also provides an opportunity to evaluate patient selection criteria for septoplasty. This NAIROS sub‐study aimed to explore the baseline relationships between clinical examination findings and objective nasal patency measures, at entry into the trial.

The study objectives were to explore:

- The baseline relationship between side of septal deflection and nasal partitioning ratio (NPR) (i.e., does the clinician stated side of septal deflection relate to the sidedness of airflow identified by the rhinospirometer?)
- The baseline relationship between appropriateness for IT reduction with PNIF measurements (the theory being that nasal decongestant reduces blood flow to the mucosal tissue, reducing the volume of the IT—does the clinician stated need for IT reduction relate with the changes observed in pre‐ and post‐decongestion PNIF?)
- The baseline relationship between observer rated airway block (ORAB) with (a) PNIF and (b) NPR measurements (can PNIF and NPR measures be used to quantify the airway block and does this relate with clinical assessments?)
- The baseline relationship between DOASS with (a) ORAB, (b) NPR and (c) side of septal deviation.

## 2 Materials and Methods

The NAIROS trial was a multicentre randomised control trial across 17 NHS hospitals in the United Kingdom [11, 12]. The clinical trial was independently funded by the National Institute for Health and Care Research Health Technology Assessment programme (NIHR HTA—reference 14/226/07). The full details of the NAIROS trial protocol were published by Rennie et al. [12]. The strengthening the reporting of observational studies in epidemiology (STROBE) reporting guideline was followed in this study.

### 2.1 Participants

Participants were 18 years or older. Participants referred to otolaryngology clinics with nasal obstruction secondary to septal deviation, confirmed on nasendoscopy and a Nasal Obstruction and Symptom Evaluation (NOSE) score [13] of 30 or more were eligible to be included in this study. Participants who had a history of previous septal surgery or systemic inflammatory disease with potential sinonasal manifestations were excluded from this study. A full list of inclusion and exclusion criteria of the NAIROS clinical trial is published Carrie et al. [11].

### 2.2 Clinical Examination

All participants were assessed at baseline with anterior rhinoscopy and nasendoscopy. The examiner assessed the following: the side of septal deviation (left, right or both), area of septum deflecting into the airway (anterior, posterior, superior, inferior, all or none), enlargement of IT (‘yes’ or ‘no’, if the IT required reduction) and ORAB (more than 50% or less than 50% blockage due to septal deviation).

### 2.3 Objective Nasal Patency Measures

At baseline, participants underwent PNIF and rhinospirometry. Participants were acclimatised for 20 min in a room with stable temperature and humidity prior to these measurements. The measurements were performed pre‐ and post‐nasal decongestant. Two sprays of 0.1% xylometazoline hydrochloride nasal spray were used in each nostril to decongest the nose. The post‐decongestant measurements were measured between 5 and 60 min post‐decongestant.

PNIF measures nasal inspiratory flow during a forced sniff manoeuvre through both nostrils, simultaneously. PNIF was measured using the PNIF meter (GM Instruments, Kilwinning, UK) with a single‐patient‐use anaesthetic face mask. Three measurements of PNIF (L/min) were performed before nasal decongestant was applied and three measurements performed following decongestant. The highest PNIF value was recorded out of three measurements. The signed PNIF change (i.e., either ±) was calculated (post‐decongestant PNIF minus pre‐decongestant PNIF).

Rhinospirometry was performed using the NV1 Rhinospirometer; (GM Instruments, Kilwinning, UK). External nosepieces were used and both sides had single‐patient‐use bacterial viral filters in line. The rhinospirometry simultaneously measures flow through the right and left sides separately.

Rhinospirometry measurements were performed pre‐ and post‐nasal decongestant: Measurements include ×3 maximal inhalation rate (mL/s) (i.e., slow inhalation of the full capacity of the lungs through both nostrils).

Pre‐ and post‐decongestant NPR were calculated using the following formula [14]:

$$\begin{array}{c} \frac{\text{(Left sided maximal inhalation rate}-\text{right sided maximal inhalation rate)}}{\text{(Left sided maximal inhalation rate}+\text{right sided maximal inhalation rate)}}. \end{array}$$

A total of three separate measurements of maximal inhalation rate was measured pre‐ and post‐nasal decongestant. Individual NPRs for each maximal inhalation rate were calculated using the formula above. The mean NPR was then calculated using individual NPR values. NPR score ranges from −1 to +1 whereby a score of 0 indicates symmetrical nasal air flow, a score of +1 indicates complete unilateral left‐sided airflow and −1 indicates complete unilateral right‐sided airflow. [14].

### 2.4 The Double Ordinal Airway Subjective Scale (DOASS)

The DOASS is a subjective score which compares the left and right‐sided nasal patency [14]. Each nostril is rated by the patient with a score 1–10; higher scores indicate better airflow. The DOASS score at baseline was calculated using the following formula, which is analogous to the NPR calculation:

$$\frac{\left(\text{left sided score}-\text{right sided score}\right)}{(\text{left sided score}+\text{right sided score}).}$$

Scores range from −1 to +1 whereby a score of 0 indicates symmetrical nasal air flow and a score of +1 or −1 indicates complete unilateral left‐ or right‐sided airflow. The DOASS score was calculated post‐nasal decongestant.

### 2.5 Statistical Analysis

Statistical analysis was performed using SPSS, version 28.0 (IBM SPSS, Chicago, IL, USA). Descriptive statistics were reported using numbers (*N*), mean, standard deviation (SD), median (IQR) and range. Pearson correlation test, Spearman rank test and Pearson point‐biserial correlation were used to establish co‐relation. The strength of correlation is reported as described by Hensch and Evans [15]: ‘very weak’ (0.00–0.19), ‘weak’ (0.20–0.39), ‘moderate’ (0.40–0.59), ‘strong’ (0.60–0.79) and ‘very strong’ (0.80–1.0). Comparison of variables was performed using independent sample *t*‐test and one‐way Anova for parametric data and Mann–Whitney *U*‐test for non‐parametric data. A two‐sided significance level of 0.05 was used throughout.

## 3 Results

### 3.1 Participant Demographics

A total of 378 participants were recruited between January 2018 and December 2019. Participant demographic details are outlined in Table 1. The mean NOSE score was 70.6 (SD 17.4).

**TABLE 1:** Participant demographics.

Total number of participants (*N*)	378
Gender [*N* (%)]	
Male	253 (66.9)
Female	125 (33.1)
Median age (IQR)	38 years (28–50 years)
Ethnicity [*N* (%)]	
White	334 (88.4)
Asian (of Indian, Pakistani, Bangladeshi ancestry)	27 (7.1)
Other ethnic origin	12 (3.7)
Other Asian	3 (0.8)
Missing data	2 (0.5)

### 3.2 PNIF and Rhinospirometry Measurements Pre‐ and Post‐Decongestant

Baseline measures are displayed in Table 2. The median nasal decongestant time was 10.0 min (IQR 7.0–13.0 min).

**TABLE 2:** Rhinospirometry and peak nasal inspiratory flow (PNIF) measurements pre‐ and post‐nasal decongestant at baseline in 378 patients.

Pre‐decongestant NPR measurements	
Mean NPR (range)^a^	0.03 (−1 to +1)
NPR < 0, *N* (%)	186 (49.2)
NPR > 0, *N* (%)	192 (50.7)
Post‐decongestant NPR measurements	
Mean NPR (range)^a^	−0.01 (−1 to +1)
NPR < 0, *N* (%)	193 (51.1)
NPR > 0, *N* (%)	185 (48.9)
PNIF pre‐ and post‐decongestant	
Mean pre‐decongestant PNIF score, L/min (SD)	93 (43)
Mean post‐decongestant PNIF score, L/min (SD)	100 (49)
PNIF change (post‐decongestant—pre decongestant)	
Negative PNIF change, *N* (%)	94 (24.9)
Positive PNIF change, *N* (%)	223 (59.0)
No PNIF change, *N* (%)	61 (16.1)

Abbreviations: L: litres, N: number, NPR: nasal partitioning ratio, SD: standard deviation.

^a^ Mean nasal partitioning ratio ranges from −1 to +1. NPR is calculated using maximal inhalation rate. Score of 0 indicates symmetrical nasal air flow whereas a score of +1 indicates complete unilateral left‐sided airflow and −1 indicates complete unilateral right‐sided airflow.

The NPR and PNIF results are displayed in Table 2. A total of 223 participants (59%) had a positive PNIF change (i.e., post‐decongestant PNIF—pre‐decongestant PNIF), suggesting nasal decongestant improved nasal air flow.

Both pre‐decongestant and post‐decongestant mean NPR values approximate to 0 (0.03 vs.—0.01) (Table 2) indicating there was no predominance to flow through left or right nasal airways across the study population. Given similar pre‐ and post‐decongestant NPR values, for simplicity of data presentation, only pre‐decongestion NPR values will be presented to evaluate the study objectives outlined in the methods section.

### 3.3 DOASS

The mean left‐sided nostril score was 5.80 ± 2.71 and the mean right‐sided nostril score was 5.69 ± 2.79. The mean baseline DOASS score is 0.013 ± 0.435.

Clinical examination findings on pre‐operative nasendoscopy are displayed in Table 3.

**TABLE 3:** Clinical examination findings on pre‐operative nasendoscopy.

Number of inferior turbinates considered appropriate for reduction [*N* (%)]	
Yes	84 (22.2)
No	282 (74.6)
Uncertain	12 (0.3)
Side of septal deviation [*N* (%)]	
Right	146 (38.6)
Left	157 (41.5)
Both right and left	75 (19.8)
Number of enlarged turbinates [*N* (%)]	
Right	100 (26.5)
Left	92 (24.3)
Both	99 (26.2)
No enlargement	87 (23.0)
Nasal blockage [*N* (%)]	
Less than 50%	105 (27.8)
More than 50%	273 (72.2)

### 3.4 NPR and DOASS for Different Sub‐Sites of Septal Deviation

The number of participants with different sub‐sites of septal deviation, as identified by the clinicians; all unilateral septal deviation (i.e., anterior, posterior, superior and inferior deviation) was 87, unilateral anterior deviation was 144, unilateral posterior deviation was 32, unilateral inferior deviation was 29, unilateral superior deviation was 15, bidirectional deviation was 68 and no septal deviation bilaterally was 3. The mean NPR for all, anterior, posterior, inferior, superior, bidirectional and no septal deviation are 0.50, 0.55, 0.41, 0.40, 0.41, 0.40 and 0.53, respectively.

The mean DOASS for all, anterior, posterior, inferior, superior, bidirectional and no septal deviation were 0.38, 0.40, 0.37, 0.37, 0.44, 0.26 and 0.15, respectively.

### 3.5 Objective I: Relationship Between Side of Septal Deflection and Mean NPR

**FIGURE 1:**
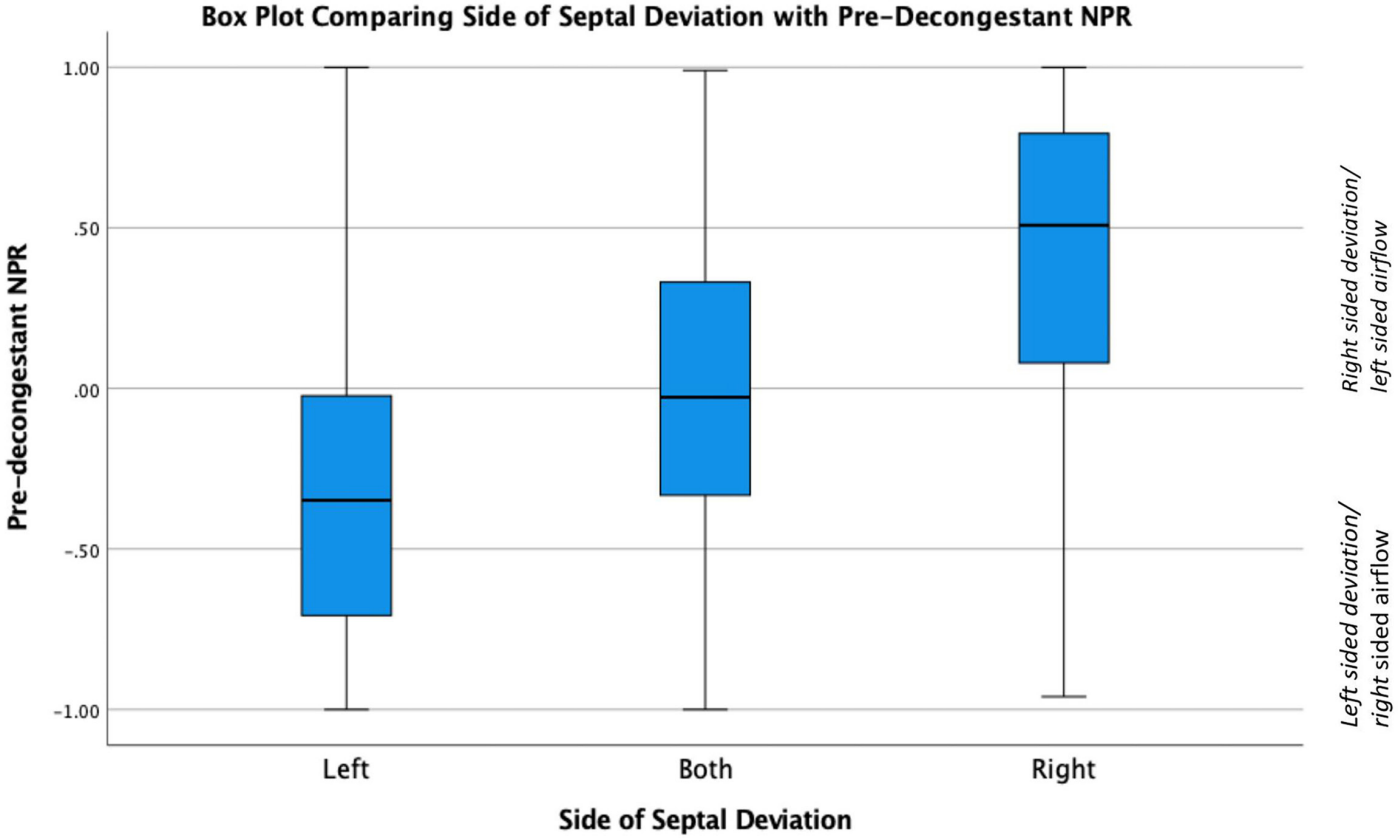
Box plot displaying the spread of pre‐decongestant NPR values based on the side of septal deviation. Black middle line in the box plot represents median and the lower and upper edges of the box represents the first quartile (25%) and third quartile (75%) data points.

Figure 1 illustrates the spread of mean NPR values based on the side of septal deviation (e.g., right‐, left‐ or both‐sided septal deviation) identified on nasendoscopy. The mean NPR for the three groups of participants defined by clinical left‐sided, both‐sided and right‐sided septal deviation was −0.35, −0.02 and 0.51, respectively (*p* < 0.001 One‐Way Anova test).

### 3.6 Objective II: The Relationship Between Appropriateness for IT Reduction With PNIF Measurements

The mean PNIF change (post‐decongestant PNIF—pre‐decongestant PNIF) for participants where the clinician did not consider the IT required reduction was 3.23 (SD ± 25.0) and mean PNIF change for participants where the clinician considered the IT did need reducing was 11.33 (SD ± 30.01), *p* < 0.01 on independent sample *t*‐test.

#### 3.6.1 Correlation Between Appropriateness for IT Reduction With PNIF Measurements

The correlation between clinician determined appropriateness for IT reduction on nasendoscopy examination with PNIF change was very weak 0.13 (*p* < 0.01, Spearman's rank correlation).

### 3.7 Objective IIIa: Relationship Between ORAB and PNIF Measurements

The mean (SD) PNIF for participants with airway block rated less than 50% was 94 (45) L/min and for participants with airway block rated more than 50% was 93 (± 42) L/min. No significant difference in mean PNIF was noted between the groups of participants with airway block rated less than 50% and more than 50%, (*p* = 0.94 on independent sample *t*‐test).

### 3.8 Objective IIIb: Relationship Between ORAB and NPR Measurements

The mean of the absolute value of NPR (regardless of sidedness) for participants with an ORAB less than 50% was 0.41 (SD 0.31). The mean NPR for participants with an ORAB more than 50% was statistically higher at 0.51 (SD 0.31) (*p* = 0.002, Mann–Whitney *U*‐test).

### 3.9 Objective IVa: Relationship Between DOASS With ORAB

The mean DOASS score (regardless of sidedness) for participants with an ORAB less than 50% was 0.33 (SD 0.23) and for participants with an ORAB more than 50% was 0.38 (SD 0.24); (*p* = 0.868—independent sample *t*‐test).

### 3.10 Objective IVb: Relationship Between DOASS With NPR

**FIGURE 2:**
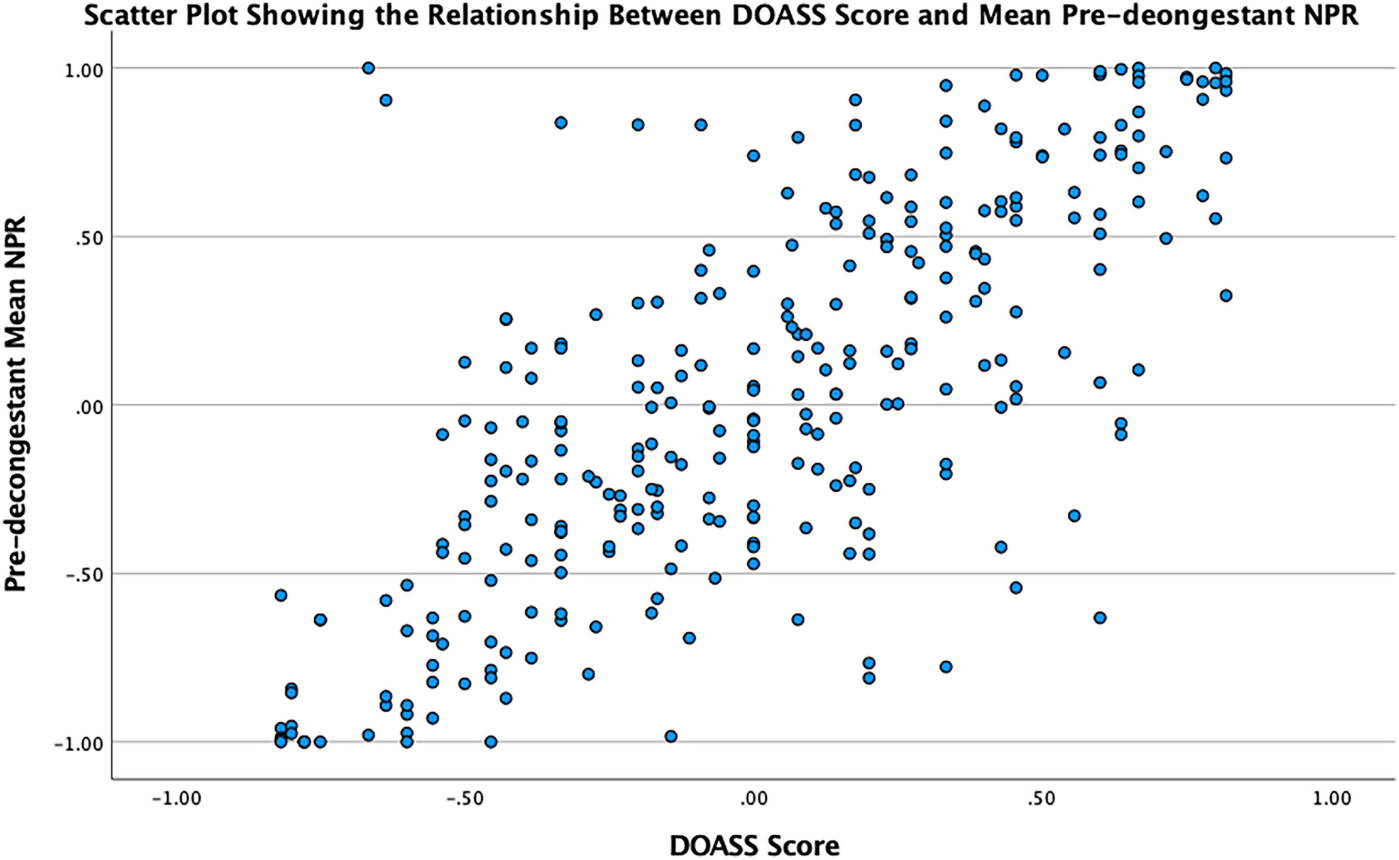
Scatter plot illustrating the correlation between signed pre‐decongestant NPR and DOASS score. DOASS score of 0 indicates symmetrical nasal air flow whereas a score of +1 indicates complete unilateral left‐sided airflow and −1 indicates complete unilateral right‐sided airflow. Similarly, NPR of 0 indicates symmetrical nasal air flow whereas a score of +1 indicates complete unilateral left‐sided airflow and −1 indicates complete unilateral right‐sided airflow.

There was a strong correlation found between the DOASS score and NPR +0.737 (*p* < 0.001, Pearson correlation test) (Figure 2).

### 3.11 Objective IVc: Relationship Between Clinician Related Rated Sidedness of Septal Deviation With DOASS Score

**FIGURE 3:**
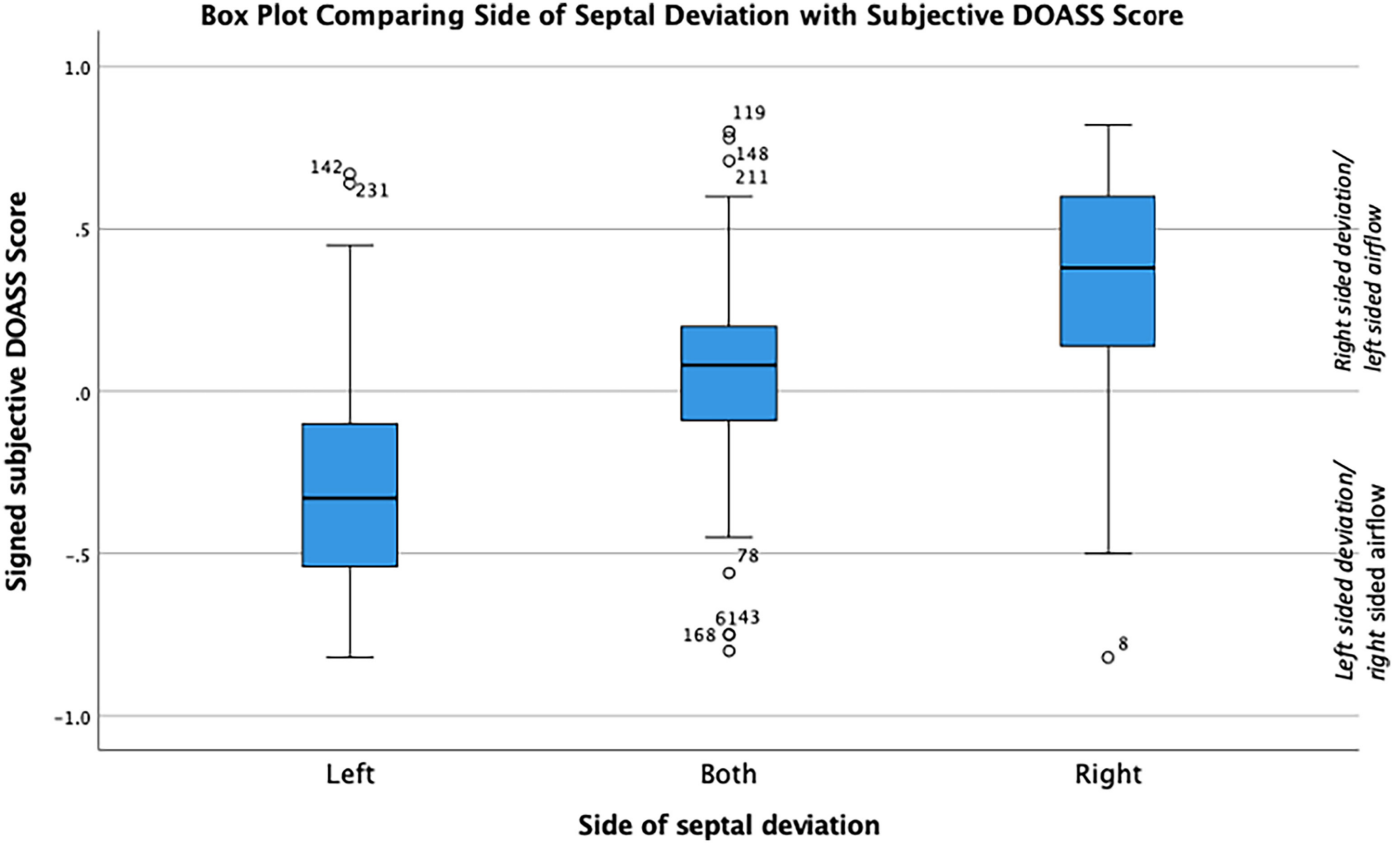
Box plot illustrating the relationship between the side of septal deviation with DOASS score.

Figure 3 illustrates the spread of the DOASS scores based on the side of septal deviation (e.g., right‐, left‐ or both‐sided septal deviation) identified on nasendoscopy. The mean DOASS score for right‐sided, both‐sided and left‐sided septal deviation participant groups was 0.32, 0.05 and −0.29, respectively (*p* < 0.001 one way ANOVA test).

## 4 Discussion

Objective nasal patency measures can assist in the understanding and diagnosis of nasal airflow disorders. At present, controversies exist as to which objective nasal patency measures are best suited for this role. This NAIROS sub‐study is the largest series exploring the baseline relationship between clinical examination findings and objective nasal patency airflow assessment in patients with nasal obstruction and septal deviation.

This study highlights that NPR values identified using rhinospirometer correlate strongly with the side of septal deviation identified by the clinician on clinical examination. Similarly, the DOASS score correlates with the side of septal deviation identified on clinical examination using nasendoscopy. The DOASS score also correlates positively with NPR and this result highlights that both the DOASS score and NPR have a high clinical utility to predict the side of nasal obstruction and septal deviation. The DOASS scoring system can be administered easily in a clinical setting when assessing a patient with nasal obstruction [14]. The DOASS score has a sensitivity of 81% with a specificity of 60% when compared to objective rhinospirometry measurement whereas clinical examination of septal deviation had previously been reported to have a sensitivity of 100% with a specificity of only 30% [14]. Boyce and Eccles highlighted that the DOASS score correlates well with rhinospirometry assessment [14] and our results further supports the role of DOASS in identifying the side of nasal obstruction and septal deviation.

In theory, enlarged IT should reduce in size following nasal decongestant and this study highlights those participants with an enlarged IT on clinical examination had a greater change in PNIF between pre‐ and post‐nasal decongestant compared to participants where IT were felt not to be enlarged on nasendoscopy. However, PNIF change (i.e., the difference between post‐decongestant PNIF and pre‐decongestant PNIF) very weakly correlated with IT enlargement on clinical examination. This measure is unlikely to offer utility to guide clinicians in decision making regarding IT reduction is appropriate. In our study, the mean change in PNIF for participants where the clinician did not consider the IT required reduction was 3.23 and mean PNIF change for participants where the clinician considered the IT did need reducing was 11.33. Timperley et al. reported that the minimum clinically important difference (MCID) (i.e., the smallest change of PNIF experienced by a patient group dement clinically significant) was 20 L/min in patients undergoing open septorhinoplasty. In the NAIROS population, reduction in PNIF did not meet previously described MCID. This may have been a reflection of the patient population recruited to NAIROS in having low rates of mucosal inflammatory disease. The difference in PNIF in NAIROS was also measured between pre‐ and post‐nasal decongestant whereas the PNIF change reported by Timperly et al. was measured in the pre‐nasal decongestant setting before and after open septorhinoplasty.

This study highlights that PNIF and DOASS are not predictive of ORAB on clinical assessment. In our study, the mean PNIF for participants in our cohort is lower than the normative PNIF [16, 17, 18], implying a dominant side to nasal breathing secondary to nasal obstruction. PNIF generally has a low clinical utility in quantifying the degree of airway block. The PNIF assessment is bilateral in nature and rapid inspiration during PNIF assessment may result in nasal valve and ala collapse causing some degree of airway block. The ORAB assessment should also be interpreted with caution as it is highly dependent on the assessor and does not take into account the degree of blockage in the posterior nasal airway. PNIF and ORAB (i.e., more than 50% or less than 50%) can be subjected to measurement bias and the assessment of ORAB in bilateral septal deviation may be less reliable when compared to unilateral septal deviation/obstruction.

The evidence on the use of objective nasal airflow measures in supporting clinical examination findings in routine clinical practise for predicting nasal obstruction is limited and inconclusive. Objective nasal airflow measures are rarely used in clinical practise other than as a research tool in the United Kingdom. Previous studies highlighted that objective techniques such as rhinomanometry assessments correlate poorly with septal deviation on rhinoscopic examination [19, 20], limiting its use to inform patients with nasal obstruction considering septoplasty. Welkoborsky et al. highlighted that airflow decreased significantly on rhinomanometry in patients with severe nasal obstruction but correlation between mild and moderate nasal obstruction identified on endoscopic examination remains low [21]. Similarly, Huygen et al. highlighted that rhinomanometric evaluation is predictive of severe nasal obstruction but its use in patients with mild nasal obstruction is poor, limiting its use in clinical practise [22]. Rhinomanometry is also invasive, time‐consuming and requires a high degree of patient cooperation to yield accurate results. The use of rhinomanometry in a very congested nose is also unreliable.

There is limited evidence on the use of rhinospirometry in the pre‐operative setting to identify the side of septal deviation. Frympas et al. reported that the correlation between side of septal deviation and NPR in patients with normal NPR is weak, but for patient with NPR outwith the normal range, the correlation between NPR and side of septal deviation is strong [23]. Boyce and Eccles reported that the correlation between NPR and side of septal deviation is very strong [14]. The decision to offer patients a septoplasty should be based on history and clinical examination using rhinoscopy and these can be further supported by patient reported outcomes such as the DOASS [14] and rhinospirometry assessments of the obstructed nasal cavity [14]. The NAIROS study showed that the main determinant of the primary outcome SNOT‐22 was the presenting level of symptoms recorded using the NOSE score. There was no difference in primary outcome SNOT‐22 scores between those participants who underwent ITR and those who did not. Although objective nasal patency measures such rhinospirometry may provide information to support clinical examination finding, the use of rhinospirometry in isolation remains debatable in clinical practise.

Nasal airflow is greater in one nostril when compared to the other at any one point due to transient nasal obstruction caused by nasal erectile tissue. The alternating physiological nasal obstruction known as nasal cycle influences the assessment of nasal obstruction to a certain degree. Factors such as the dominance of the nostril, cycle duration, breathing patterns (e.g., shallow vs. deep breathing) and body posture affects nasal cycle differently. The effect of nasal cycle should be considered by clinicians when assessing nasal obstruction and it is also important to note that the nasal cycle may influence the measurements of objective nasal patency measure at any one point [24].

One limitation is that the NAIROS trial recruited patient with a NOSE score of more than 30 (i.e., moderate, severe and extreme nasal obstruction), and only 18% of the cohort has moderate nasal obstruction (scores 30–50). The majority of participants had severe and extreme nasal obstruction and the results of this study must be interpreted with caution for patients with moderate or mild nasal obstruction. Due to the nature of NPR calculation, individuals with bidirectional septal deviation have NPR values approximating to zero, making NPR a less useful assessment when differentiating a straight septum from a bidirectionally deviated septum.

Our study also showed that 24.9% of participants had a negative PNIF change despite the use of nasal decongestant and this may be due to low rates of mucosal inflammatory disease in the NAIROS population, measurement errors, leakage of air through PNIF, patient fatigue and inappropriate positioning of PNIF mask [25]. The reproducibility and inter‐rater variability of PNIF and rhinospirometry measurements were also not considered in this study and this may impact the results of this study negatively.

In this study, septal deviation was classified as all, anterior, posterior, superior, inferior, bidirectional or none. This classification maybe subjected to observation bias and a high degree of variation in the type of deviation reported by clinicians. Subgroup analysis of the different deviation sub‐sites was not performed. Deviation of septum at the nasal valve area may also influence nasal airflow and this was not considered when classifying septal deviation. For example, severe mid‐zone septal deviation (e.g., >50%) may result in less severe nasal obstruction when compared when compared to less severe deviation (e.g., 25%) at the nasal valve area.

It is also important to note that variability in clinicians rating, subjective patient reported symptoms and objective nasal patency measures may influence the results of this study. For example, despite a strong correlation between DOASS and side of septal deviation on clinical examination, a small proportion of patient reported DOASS were non‐concordant with the side of septal deviation. Although the mean NPR scores shows a moderate to strong correlation with the side of septal deviation, it is important to note that the mean NPR does not match perfectly with the side of septal deviation. For example, the mean NPR for right‐sided septal deviation is only 0.51 as opposed to a perfect agreement of 1. The results of this study should be interpreted with caution as no perfect match between patients' perceptions, clinician perceptions and objective measurements exist.

The role of objective nasal patency measures in identifying patients who report positive outcomes from septoplasty requires further analysis. The NAIROS study found that no objective nasal patency measures were related to the primary outcome (i.e., the SNOT‐22 at 6 months) [11] and further in‐depth analyses are planned.

## 5 Conclusion

This study identified strong relationships between the clinician rated side of septal deflection, the patient reported DOASS and the objective NPR measurements. NPR and the clinician rated degree of airway blockage were concordant. The relationships between the PNIF measurements and the clinical examinations did not appear to be of clinical utility in this population.

## Author Contributions

Pavithran Maniam is the lead author and was involved in conceptualisation of research question, analysis of data and writing of this manuscript. Alison Bray was involved in data collection and writing of this manuscript. Michael Drinnan was involved in the project and provided expert statistical advice and opinion on the research. Tony Fouweather was involved in data analysis and provided expert statistical advice and opinion on the research. M. Dawn Teare provided expert advice and opinion on the research. Sean Carrie was the project supervisor, NAIROS clinical trial lead and was involved in writing of this manuscript. James O'Hara was the project supervisor, NAIROS clinical trial lead and was involved in writing of this manuscript.

## Ethics Statement

This study is a post hoc analysis of the NAIROS trial. Ethical approval for the NAIROS trial: The North East—Newcastle and North Tyneside 2 UK Health Research Authority Regional Ethics Committee (August 2017, study reference No 17/NE/0239).

## Conflicts of Interest

The authors declare no conflicts of interest.

## Data Availability

The data that support the findings of this study are available from the corresponding author upon reasonable request.
